# The Medico-Legal Aspects of Poisons—I

**Published:** 1909-07-31

**Authors:** 


### Medico-Legal Points.

### THE MEDICO-LEGAL ASPECTS OF POISONS—I.

### Definition of a Poison.

The means of ascertaining the traces of poisons, either on the living or the dead body, is one of the most important subjects in legal medicine, and its importance is only equalled by its difficulty. What is a poison?

The ancients considered everything as poisonous that produced malignant symptoms, and attacked what may perhaps be styled the vital principle. The common ideas of poison by the moderns, on the other hand, is that it is a substance which, on being applied in one or other way to the human body, is capable of destroying the action of the vital functions. It is difficult, however, to give a definition to the term which will meet the signification attached to it by different classes of persons; for while, in common language, poisons are understood to be those articles only which are deadly in small doses—as strychnine, prussic acid, arsenic, etc.—the lawyer and the physician will unite in affixing to it a much wider meaning.

Every medical practitioner is aware that very many active remedial substances, which are not injurious when taken in ordinary amounts, may, in an overdose, produce serious and fatal effects. Moreover, questions may arise as to the applicability of the term to substances which destroy life by mechanical irritation, such as powdered glass, etc., although the administration of such substances to another, with intent to injure, is made a criminal offence in the various penal codes. Section 11 of the 24 and 25 Vict. c. 1 enacts that "Whoever shall administer, or cause to be administered to, or taken by any person, any poison or other destructive thing, with intent to commit murder, shall be guilty of felony." The French law interdicts the wilful administration of any substances "which, without being of a nature to produce death, are injurious to health."

### Legal Quibbles.

Chief Justice Cockburn, in Reg. *v.* Henna (Cornwall Lent Assizes, 1887), supported the ingenious contention of the defence "that there must be a distinction between a thing only noxious when given in excess, and a thing which is a recognised poison and is known to be a thing noxious and pernicious in its effects; that unless a thing was noxious in the quantity administered, it cannot be said that there has been a noxious thing administered." Passing over the discussion as to whether cantharides, used in the case from which the above quotation is taken, is primarily a poison because it is placed in the schedule of the English Act for regulating the sale of poisons, such as strychnine, prussic acid, etc., we are more concerned with the principle of what constitutes a poison than whether the dose of the substance administered is sufficient to cause death. If the definition of a poison is dependent upon the quantity administered, as an established fact in medical jurisprudence, the present list of poisonous substances must be modified by a statement of the dose of a substance which makes the substance act as a poison.

It would appear to be much better to leave to the competent medical expert, who should be abreast of the times with the known results of modern technical sciences to inform in each case brought for trial or judicial inquiry whether the quantity of the substance supposed to have caused death would ordinarily have been injurious to bodily health, and in what way it may have brought about the death. If such a method should be followed less confusion would result than from a prolonged discussion upon the meaning and play of words and terms.

### Immunity to Poisons.

Cases in which a really poisonous substance has been taken with impunity are more rare. In the majority the immunity is only comparative, the person being affected merely in a less degree than is usual. This important consideration must not be overlooked, for there are many cases recorded in which, for instance, among other deleterious medicines, opium, arsenic, belladonna, digitalis, chloral hydrate, and even strychnine, have been taken with impunity in amounts usually considered fatal to mankind. It follows, therefore, that the use of definite words or phrases to restrict or confirm a schedule of poisons as a medico-legal requirement is in general inadvisable. The medical expert should be well qualified to explain clearly to the court in what manner the substance in a given manner may, or usually does, destroy life, and the amount of it required to be in contact with the tissues, the life and functions of which when alive are shown by him to have been destroyed.

The remarkable resistance that is sometimes observed to the action of poisons deserves notice. Among the Hungarians the seeds of the *Palma Christi* are often taken to the amount of thirty-six grains without any inconveniences, and some of the French peasantry use a decoction of *colocynth* as a common purgative. The common dose of the extract of *aconitum napellus* is one or two grains, and it is deemed dangerous to use it in large quantities; but Foderé was consulted concerning the case of Charles IV. of Spain, who, while residing at Marseilles, was attacked with rheumatic gout, and he recommended the medicine in question. M. Soria, the king's physician, replied that at a former period it had been administered for a length of time, and to such an extent that the patient took a drachm daily without any good or evil effects (Foderé, vol. iii. p. 463).

The fumes of mercury, of lead, and of copper are well known to be injurious to those who inhale them; yet no fact is better established than that of workmen resisting their effects for many years. Such extraordinary instances should, above all, never influence us in legal medicine, nor lead us to the idea that because one person has taken a particular substance without any ill effects, it is, therefore, not a poison.

Differences between Man and Lower Animals.

There is another curious fact connected with this subject which it is proper to mention. It is the different effects which some substances produce on man and other animals, being noxious to the one and innoxious to the other, and *vice versa*. Thus, sweet almonds are said to kill dogs, foxes, and fowls; aloes are destructive to dogs and foxes; pepper to hogs; and parsley to the parrot. On the contrary, hogs feed on henbanes, pheasants on stramonium, and goats on water-hemlock with impunity. Many, however, of the principal poisons produce similar results on man and other animals, and in none, probably, is the resemblance greater than with the dog. The rapidity of the action of poisons varies considerably. Concentrated hydrocyanic acid destroys an adult man almost in an instant, whilst others take away life within an hour, a few hours, a day, or a longer period. Some, indeed, when the sufferer escapes the immediate consequences, prove fatal after months or a year, but with a sufficiently marked train of symptoms to indicate with certainty the original cause. It is on this account that no particular period has been introduced in the laws of some countries; and, if the poisoned person dies the criminal is to suffer punishment.

Ptomaine Poisoning.

The idiosyncrasy which converts a harmless substance into a poisonous agent is very frequently observed in the case of articles of food. Thus mussels, fish, pork, and mutton have frequently given rise to all the symptoms of irritant poisoning. It should be remembered, however, that the symptoms in these cases may result as well from the mechanical irritation of the food—too large a meal having been taken—or from the fact of its being in a condition unfit for use; while some meats become poisonous because they are infested with animal parasites, or from commencing putrefaction. There is another source of food poisoning which should not be overlooked; we refer to the meat which is sold in tin cans.

If in preparing these canned meats care is not taken to drive out the atmospheric air by thoroughly boiling the liquid contained in them, there may occur a decomposition which will produce an injurious substance. This may not necessarily cause death, though it does commonly produce serious gastro-intestinal disturbances, such as diarrhœa and colicky pains, which may simulate a diseased condition. It was formerly supposed that this train of symptoms was due to the action of the zinc and lead compounds used in soldering the cans, but it is more likely caused by products of fermentation processes, which are favoured by the contained air. It should be remembered that these deleterious substances cannot always be readily recognised by taste and smell. The medical expert should be qualified to recognise the micro-organisms by the aid of the microscope and their propagation in cultures.

The Factors of Dose and Disease.

The quantity of any poison which may cause injury to the living tissues in the human body and destroy their functions of life is dependent, not so much upon the amount of the substance which is administered by the mouth, as by the amount which is absorbed. This is also dependent on the rapidity of the elimination after the ingestion. If the substance is removed very rapidly by vomiting or diarrhœa, and without causing serious injury to the tissue of the alimentary canal, an amount may remain to be absorbed by the blood insufficient to produce a poisonous action upon the general system.

Disease also has sometimes the effect of rendering the system tolerant of substances which would be poisonous in the same doses in a healthy state of the system. In acute alcoholism, or alcoholic intoxication, the blood and tissues may be so loaded with alcohol as to interfere wholly or partially with the absorption of a poisonous agent. During the active stage of severe inflammatory and febrile diseases, mercury may be given in large and repeated doses without producing salivation.

